# Supplementary material for: Status of Insecticide Resistance and Its Mechanisms in Anopheles gambiae and Anopheles coluzzii Populations from Forest Settings in South Cameroon
Source: Genes (Basel). 2019 Sep 24;10(10):741. doi: 10.3390/genes10100741 (PMC6827028; doi:10.3390/genes10100741)
Supplement: Supplementary file 1 [file genes-10-00741-s001.zip › genes-575546-supplementary/Figure S1.docx]

**Supplemental information**

**Figure S1** Incidence of resistance alleles in different populations of *An. gambiae* mosquitoes (Part A: kdr L1014F and L1014S, Part B: kdr N1575Y, Part C: iAChe G119S mutant allelic frequencies). Images were created using the Google My Maps application.
