# Supplementary material for: Status of Insecticide Resistance and Its Mechanisms in Anopheles gambiae and Anopheles coluzzii Populations from Forest Settings in South Cameroon
Source: Genes (Basel). 2019 Sep 24;10(10):741. doi: 10.3390/genes10100741 (PMC6827028; doi:10.3390/genes10100741)
Supplement: Supplementary file 1 [file genes-10-00741-s001.zip › genes-575546-supplementary/Figure S2 Gene expression analysis in the five populations of the study.docx]

**Figure S2** Gene expression analysis in the five populations of the study (Parts A-E) compared to the two susceptible laboratory strains (Kisumu, Ngousso). Error bars indicate 95% CIs. * indicates genes with statistically significant upregulation. ▲indicates genes with consistent upregulation compared to both susceptible strains.
