# Supplementary material for: Status of Insecticide Resistance and Its Mechanisms in Anopheles gambiae and Anopheles coluzzii Populations from Forest Settings in South Cameroon
Source: Genes (Basel). 2019 Sep 24;10(10):741. doi: 10.3390/genes10100741 (PMC6827028; doi:10.3390/genes10100741)
Supplement: Supplementary file 1 [file genes-10-00741-s001.zip › genes-575546-supplementary/Table S1 List of primers and probes used in the present study.docx]

**Table S1** List of primers and probes used in the present study

| **Oligo** | **Assay** | **Sequence (5'-3')** | **Concentration (nM)** |
| --- | --- | --- | --- |
| *RPS7*_F | Dtx (A)-(D) | CCACCATCGAACACAAAGTTGA | 100 |
| *RPS7*_R | Dtx (A)-(D) | TGCTGCAAACTTCGGCTATTC | 200 |
| *RPS7*_P | Dtx (A)-(D) | FAM-CCGTGACGTTACGTTCGAATTCCCA-BHQ1 | 250 |
| *CYP6P3*_F | Dtx (A) | ACAATGTGATTGACGAAACCCT | 400 |
| *CYP6P3*_R | Dtx (A) | GGATCACATGCTTTGTGCCG | 500 |
| *CYP6P3*_P | Dtx (A) | HEX-ACCCGCGTACCGTCTGTGGACT-BHQ1 | 350 |
| *CYP6M2*_F | Dtx (A) | CTGGCGTTGAATCCAGAGGT | 600 |
| *CYP6M2*_R | Dtx (A) | GATACTTGCGCAGTGATTCATTAAG | 400 |
| *CYP6M2*_P | Dtx (A) | ATTO647N-AGAGAAATCCTGCAAAAGCACAACGGAGA-BHQ3 | 250 |
| *CYP9K1*_F | Dtx (B) | CCGACACGTGGTGATGGATAC | 200 |
| *CYP9K1*_R | Dtx (B) | CGTCGTCGGTCCAGTCAAC | 400 |
| *CYP9K1*_P | Dtx (B) | HEX-CAATCTTCTGATGCAGGCCCGCAA-BHQ1 | 300 |
| *CYP6P4*_F | Dtx (B) | CTGGACAACGTTATCAATGAAACC | 400 |
| *CYP6P4*_R | Dtx (B) | GCACGGTGTAATCACGCATC | 500 |
| *CYP6P4*_P | Dtx (B) | ATTO647N-CCGATCGAGTCACTTTCGCGCG-BHQ3 | 300 |
| *CYP6Z1*_F | Dtx (C) | CCCGCAACTGTATCGGTCTG | 100 |
| *CYP6Z1*_R | Dtx (C) | TTCGGTGCCAGTGTGATTGA | 600 |
| *CYP6Z1*_P | Dtx (C) | HEX-TGATGCTGTCCCGATTTAACTTTTCGGC-BHQ1 | 250 |
| *GSTE2*_F | Dtx (C) | CCGGAATTTGTGAAGCTAAACC | 100 |
| *GSTE2*_R | Dtx (C) | GCTTGACGGGGTCTTTCGG | 400 |
| *GSTE2*_P | Dtx (C) | ATTO647N-CGGTACGATCATCACCGAGAGCCAC-BHQ3 | 300 |
| *CYP6P1*_F | Dtx (D) | ACAGGTGGTGAACGAAACCC | 100 |
| *CYP6P1*_R | Dtx (D) | GGTGTAATCCTGTCCCGCAA | 500 |
| *CYP6P1*_P | Dtx (D) | HEX-CCGCTCGAAACGACGCTGCG-BHQ1 | 300 |
| *CYP4G16*_F | Dtx (D) | GTCCAAGAAGTTGCGTCGGAC | 200 |
| *CYP4G16*_R | Dtx (D) | TCTTCGATTTGCGTTGACGTG | 200 |
| *CYP4G16*_P | Dtx (D) | ATTO647N-CTGCAGGCCGACATCATTTTGAAGC-BHQ3 | 300 |
| kdr_F | L1014F/S | CATTTTTCTTGGCCACTGTAGTGAT | 500 |
| kdr_R | L1014F/S | CGATCTTGGTCCATGTTAATTTGCA | 200 |
| kdr-wt(L)_P | L1014F/S | HEX-CTTACGACTAAATTTC-MGB | 500 |
| kdr-mt(F)_P | L1014F/S | FAM-ACGACAAAATTTC-MGB | 500 |
| kdr-mt(S)_P | 1014F/S | ATTO647N-ACGACTGAATTTC-MGB | 500 |
| 1575_F | N1575Y | TGGATCGCTAGAAATGTTCATGACA | 500 |
| 1575_R | N1575Y | CGAGGAATTGCCTTTAGAGGTTTCT | 200 |
| 1575-wt(N)_P | N1575Y | HEX-ATTTTTTTCATTGCATTATAGTAC-MGB | 300 |
| 1575-mt(Y)_P | N1575Y | FAM-TTTTTCATTGCATAATAGTAC-MGB | 400 |
| ACE1-F | iAChe | GGCCGTCATGCTGTGGAT | 200 |
| ACE1-R | iAChe | GCGGTGCCGGAGTAGA | 600 |
| Ace1-wt(G) _P | iAChe | HEX-TTCGGCGGCGGCT-MGB | 400 |
| Ace1-mut(S)_P | iAChe | FAM-TTCGGCGGCAGCT-MGB | 400 |
| ID_F | Species ID | GTGAAGCTTGGTGCGTGCT | 300 |
| ID_R | Species ID | GCACGCCGACAAGCTCA | 300 |
| Ag+_P | Species ID | HEX-AGCGGAACAC-MGB | 250 |
| Aq +_P | Species ID | FAM-AGCGGGACAC-MGB | 250 |
| Aa+_P | Species ID | ATTO647N-ACATAGGATGGAGAAGG-MGB | 250 |
| S200 X6.1_F | Molecular Forms | TCGCCTTAGACCTTGCGTTA | 800 |
| S200 X6.1_R | Molecular Forms | CGCTTCAAGAATTCGAGATAC | 400 |
| AgM form_P | Molecular Forms | HEX-ACCGCGCCGCCATACGTAGGA-BHQ1 | 400 |
| AgS form_P | Molecular Forms | FAM-ATGTCTAATAGTCTCAATAGT-MGB | 300 |

*Abbreviations*: Dtx: Detox F, Forward primer; R, Reverse Primer; P, TaqMan Probe
