# Supplementary material for: Status of Insecticide Resistance and Its Mechanisms in Anopheles gambiae and Anopheles coluzzii Populations from Forest Settings in South Cameroon
Source: Genes (Basel). 2019 Sep 24;10(10):741. doi: 10.3390/genes10100741 (PMC6827028; doi:10.3390/genes10100741)
Supplement: Supplementary file 1 [file genes-10-00741-s001.zip › genes-575546-supplementary/Table S3 Summary of all resistance mechanisms for each of the study.docx]

**Table S3** Summary of all resistance mechanisms for each of the study’s populations

| **Resistance Mechanism** | **Population** | | | | |
| --- | --- | --- | --- | --- | --- |
|  | **Bastos (Yaoundé)** | **Nkolondom (Yaoundé)** | **Sangmelima** | **Mbandjock** | **Nyabessan** |
|  |  |  |  |  |  |
| *Target site mutations* | | | | | |
| kdr L1014F/S |  |  |  |  |  |
| kdr L1014S |  |  |  |  |  |
| kdr N1575Y |  |  |  |  |  |
| iAChE G119S |  |  |  |  |  |
| *Metabolic and CHC biosynthesis* *gene overexpression* | | | | | |
| *CYP6P3* |  |  |  |  |  |
| *CYP6M2* |  |  |  |  |  |
| *CYP9K1* |  |  |  |  |  |
| *CYP6P4* |  |  |  |  |  |
| *CYP6Z1* |  |  |  |  |  |
| *GSTE2* |  |  |  |  |  |
| *CYP6P1* |  |  |  |  |  |
| *CYP4G16* |  |  |  |  |  |
